# Neurodevelopmental delay according to severity of deformational plagiocephaly in children

**DOI:** 10.1097/MD.0000000000021194

**Published:** 2020-07-10

**Authors:** Dong Han Kim, Dong Rak Kwon

**Affiliations:** Department of Rehabilitation Medicine, Catholic University of Daegu School of Medicine, Daegu, South Korea.

**Keywords:** Denver Development Screening Test, neurodevelopmental delay, plagiocephaly

## Abstract

The purpose of this study was to investigate the prevalence of neurodevelopmental delay among deformational plagiocephaly (DP) children, and to confirm relationship between neurodevelopmental delay and severity of DP.

This study is retrospective study. Five hundred thirteen children who visited for abnormal head shape through outpatient department were recruited. To identify the children with neurodevelopmental delay among the 513 children with DP, Denver Development Screening Test (DDST) was performed in 38 children who suspected of neurodevelopmental delay. Cranial vault asymmetry (CVA) was measured by using caliper, and cranial vault asymmetry index (CVAI) was calculated. Thirty eight children with DP who conducted DDST were divided into 2 groups according to the degree of CVA; group 1 included 21 children with CVA under 10 mm, and group 2 included 17 children with CVA over 10 mm.

There was a significant difference in number of neurodevelopmental delay between group 1 (n = 7) and group 2 (n = 14) (*P* < .05). Mean grade of DP, CVA, and CVAI (1.76 ± 0.44, 5.90 ± 2.21 mm, 4.20 ± 1.51%) in group 1 was smaller than that in group 2 (3.41 ± 0.8, 12.71 ± 3.22 mm, 8.83 ± 2.18%), respectively (*P* < .05).

Our results found that the frequency of developmental delay was significantly increased in children with CVA more than 10 mm. Doctors who take care of children with DP had better keep developmental delays in mild.

## 1 Introduction

Plagiocephaly is a term broadly referring to cranial asymmetry.^[1]^ Immature fusion of 1 or more cranial sutures may cause plagiocephaly, which is, however, more commonly caused by external forces acting on the infants cranium, such as intra-uterine constraint, assisted vaginal delivery, primiparity, prolonged labor, multiple births, male gender, unusual birth position, positional preference, torticollis, and supine sleeping position.^[2–7]^ This is called deformational plagiocephaly (DP). The incidence of DP is known to be between 1/300 and 1/10.^[8]^

Since DP has been considered to be a benign condition, few studies have been conducted on the effects of DP on childrens neurodevelopmental outcomes and it has not been clarified yet whether DP and neurodevelopmental delay have correlation each other. However, recent studies including children with craniosynostosis has reported significantly increased risk of developmental delays in these children.^[9,10]^ Collett^[11]^ et al performed a brain MRI and Bayley Scales of Infant and Toddler Development-III (BSID-III) in 41 in children with and without DP examining brain volume and shape, showing that children with asymmetric and compressed skull had the corpus callosum positioned at a greater angle and shortened when compared to the unaffected controls. In addition, the height and height-width ratio of cerebellar vermis were found to be greater in children with DP than those without DP. Accordingly, it is suggested that the difference in head shape measurement could be associated with child development. Kordestani^[12]^ et al also reported that 110 children with DP showed significant delays in psychomotor development and none of the children showed accelerated development. Also, Hutchison^[13]^ et al demonstrated that children with DP were more likely to show a decreased activity level and perceived developmental delay than healthy children. Davis^[14]^ et al reported that children with DP were shown to reach motor milestones later than non-plagiocephalic children. However, none of the previous studies have yet clarified the clear causal relationship between cranial asymmetry and neurodevelopment, remaining many controversies.^[9,15–17]^ Therefore, this study aimed to investigate the prevalence of neurodevelopmental delay among DP children, and to examine relationship between neurodevelopmental delay and severity of DP.

## 2 Methods

This study was performed after receiving approval from the Institutional Review Board and Ethics Committee at the Daegu Catholic University Hospital, in accordance with the Declaration of Helsinki.

The present study is a retrospective study conducted in outpatient clinics in Department of Rehabilitation Medicine at Daegu Catholic University Hospital between September 2010 to May 2018. Data on 513 children with confirmed plagiocephaly without craniosynostosis were retrospectively collected. It is confirmed with ultrasound on skull, around lambdoidal suture that none of the children were diagnosed with craniosynostosis. DP, also known as non-craniosynostotic plagiocephaly, refers to a craniofacial asymmetry, most commonly presented as unilateral flattening of the occiput.^[2,18]^

### 2.1 Patients

Five hundred thirteen children who visited outpatient clinics at department of rehabilitation at Daegu Catholic University Hospital for abnormal head shape were recruited. Children who met the following criteria were included: children with

1. cranial asymmetry that presents with flattening of 1 side of cranium and
2. ability to comply with caliper cephalometry and ultrasound measurements. The children who were confirmed to have craniosynostosis by ultrasound were excluded.

### 2.2 Clinical presentation

**Figure 1:**
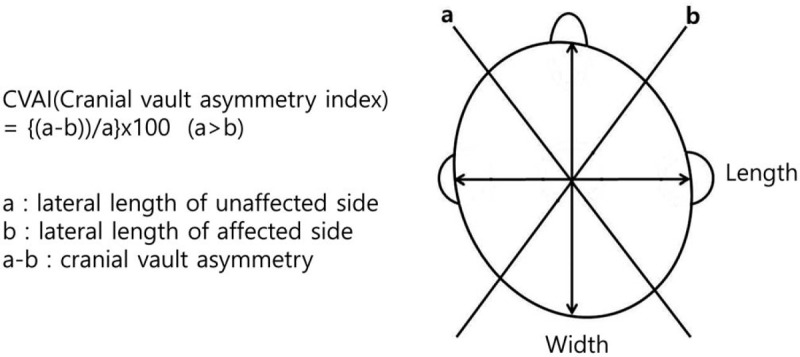
Cranial vault asymmetry index (CVAI) was calculated using caliper. The cranial diagonal diameter was measured on unaffected (a) and affected sides of cranium (a > b). Cranial vault asymmetry (CVA) is defined as the difference between the cranial diagonal diameters (a–b) divided by long cranial diagonal diameter (a); and CVAI is CVA multiplied by 100. CVA = cranial vault asymmetry, CVAI = cranial vault asymmetry index.

**Figure 2:**
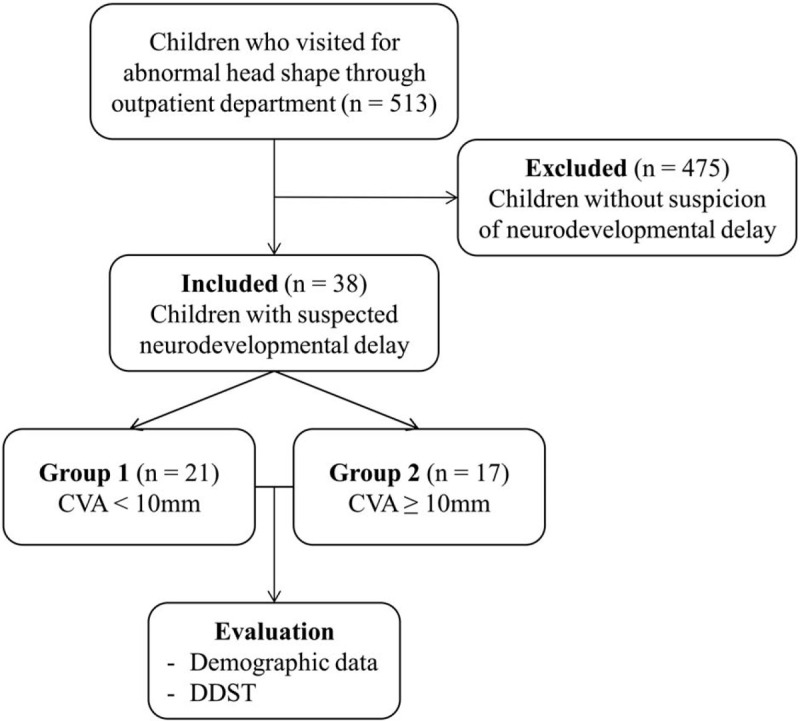
Flow diagram indicating progress of participants through the study. CVA = cranial vault asymmetry, DDST = Denver development screening test.

The weight, height, and head circumference of children were recorded on their visits. The measurement was performed by a pediatric physiatrist using caliper cephalometry, which is a simple method to examine the severity of DP and provide accurate information on the main diagnostic features of the disease.^[19,20]^ Horizontal length was defined as the distance between the contralateral occipital and frontozygomatic bones and was measured on both sides of the cranium, the unaffected (a) and affected (b). Cranial vault asymmetry (CVA) was calculated by dividing CVA (a–b) with the horizontal length of the unaffected side (a) and multiplying by 100, and cranial vault asymmetry index (CVAI) was calculated by dividing CVA (a–b) by the horizontal length of the unaffected side (a) and multiplying with 100 (Fig. 1).^[21]^ Ultrasonographic measurements were performed to exclude craniosynostosis by a physiatrist expert on performing musculoskeletal ultrasound using an EPIQ 5 (Philips Healthcare, USA) ultrasound system with a 9–4 MHz multi-frequency linear transducer. Entire skull was scanned from the mastoid fontanelle to the posterior fontanelle in all children. The transverse scanning of both lambdoid sutures was performed and short cine loop of their flattest parts of occipital bone was recorded.^[21]^ All assessments were performed by the same researcher. To identify the children with neurodevelopmental delay among the 513 children with DP, 38 children who were suspected to have neurodevelopmental delay were examined with Denver Development Screening Test (DDST) and were classified into 2 groups according to the degree of CVA; group 1 included 21 children with CVA under 10 mm, and group 2 included 17 children with CVA over 10 mm (Fig. 2). In DDST, each item consists of 4 developmental domains: personal-social, fine motor-adaptive, language, and gross motor. Each test item is scored as pass, fail, or refused. Delay defined as a child failing a test item which 90% of his or her age mates pass, and caution defined as a child failing a test item which between 75% from 90% of his or her age mates pass. We rated a childs test performance as follow: “normal” means no delay in any domain and no more than 1 caution; “questionable” means one delay or more than 2 cautions; “abnormal” means 2 or more delays.^[22,23]^ In our study, we regarded as the child to have a neurodevelopmental delay when the result of DDST was questionable or abnormal. Demographic data of these children were collected.

### 2.3 Statistical analysis

IBM SPSS ver. 19.0 (IBM Co., Armonk, NY, USA) was used for the statistical analysis with the level of significance set at *P* < .05. Data are presented as the mean ± standard deviation. Analysis on intergroup differences with respect to measured parameters was performed using the Fisher exact test. The independent *t* test was performed to compare the CVA and CVAI in both groups.

## 3 Results

There was no significant difference in demographic data containing age, gender, affected side, and risk factors between group 1 and group 2, which included 38 children who underwent DDST (Table 1). Mean grade of DP, CVA, and CVAI (1.76 ± 0.44, 5.90 ± 2.21 mm, 4.20 ± 1.51%) in group 1 was found to be smaller than those of group 2 (3.41 ± 0.8, 12.71 ± 3.22 mm, 8.83 ± 2.18%) (*P* < .001, Table 1). Six children in group 1 and 12 children in group 2 were questionable, and 1 child in group 1 and 2 children in group 2 were abnormal in DDST. There was a significant difference in number of children with neurodevelopmental delay between group 1 and group 2, 7 children among group 1, while 14 among group 2 (*P* = .003, Table 2). CVAI was found to be significantly larger in neurodevelopmental delay group (n = 21, 7.39 ± 3.24%) than in non-neurodevelopmental delay group (n = 17, 4.89 ± 1.84%) (*P* < .05). Furthermore, the demographic and clinical findings in 21 children with a diagnosis of neurodevelopmental delay in DDST are listed on Table 3.

**Table 1:**
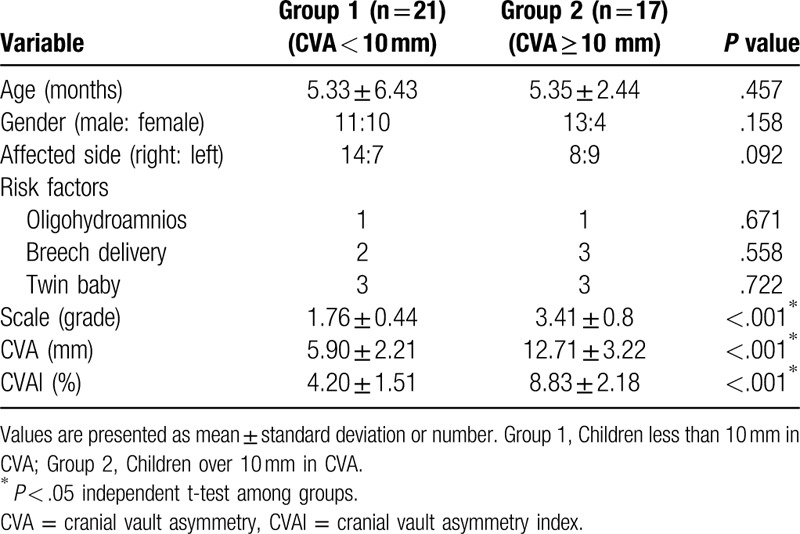
Demographic and clinical characteristics of children.

**Table 2:**
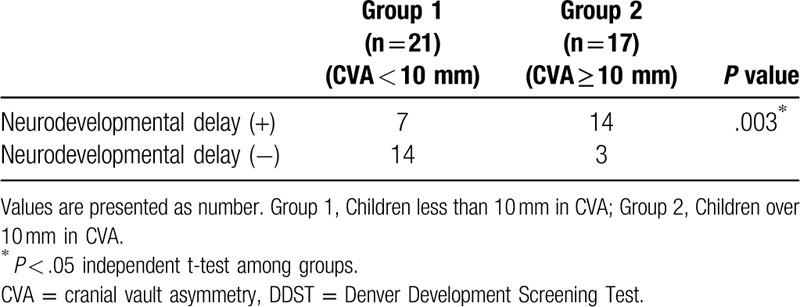
Prevalence of diagnosis with neurodevelopmental delay in DDST between group 1 and group 2.

**Table 3:**
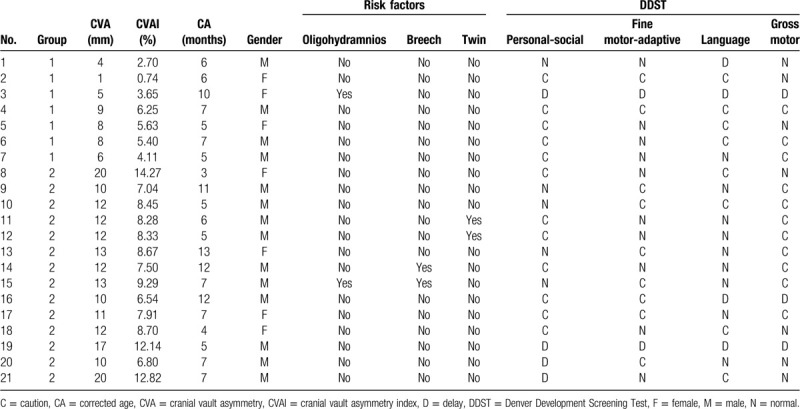
Demographic and clinical findings in 21 children with a diagnosis of neurodevelopmental delay in DDST.

## 4 Discussion

In this study, it is confirmed with ultrasound on skulls around the lambdoidal suture that none of the children were diagnosed with craniosynostosis. Unlike craniosynostotic plagiocephaly, DP is typically treated with a customized orthotic molding helmet, and is often diagnosed before the child becomes 1 year old, when the skull is malleable and still growing rapidly.^[2,4]^

In current study, severity of CVA was verified by the caliper cephalometry. Caliper cephalometry is a non-invasive and easily applicable method that provides precise information on major diagnostic features of DP.^[20]^ In previous several studies, Moss^[24]^ et al, and Mortenson^[25]^ et al defined a CVA as normal <3 mm, mild/moderate ≤12 mm, moderate/severe > 12 mm. Meanwhile, according to the cranial molding therapy protocol, children with DP were treated on the basis of a CVA cutoff value of 10 mm.^[26,27]^ Based on these previous studies, we determined that neurodevelopmental delay can be considered when the CVA is more than 10 mm, which corresponds to a severity of moderate to severe. Therefore, the criteria of CVA for dividing groups were set as 10 mm. Likewise, Argenta^[8]^ et al reported 5 stages of DP progressing from minimal to severe deformation. Type 1 is restricted to the back of the skull, while type 2 adds malposition of the affected ear and type 3 adds forehead deformity. Type 4 adds malar deformity and type 5 adds brain attempts to decompress temporally or vertically. In current study, group 1 was shown to have a mean scale of DP of 1.76, while the group 2 to have 3.41. Therefore, our results has demonstrated that grade of DP rated is significantly higher in group 2 than in group 1.

The main findings of this study showed that the incidence of neurodevelopmental delay were significantly different between in group 1 and group 2, which is consistent with previous studies.^[8]^ We hypothesized and confirmed that children with more severe DP are more likely to have neurodevelopmental delay. Collett^[11]^ et al reported that asymmetric and compressed skulls may affect brain parenchyma, leading to lower scores on cognitive and motor function in BSID-III evaluation, which may result in developmental delays. Similarly, Kordestani^[12]^ et al revealed that children with DP showed delays in both psychomotor and mental development assessed by BSID-II.

Starting with the adaptation of the Gesell development schedule by Knobloch^[28]^ et al in 1966, and the 1967, introduction of the DDST by Frankenburg^[22,23]^ et al, various developmental screening tools have been applied to millions of children. So far, the DDST is the most commonly used and thoroughly studied screening test in worldwide.^[23]^ Among 513 children with abnormal head shape who visited outpatient clinic, we performed DDST on 38 children who were suspected to have developmental delay, and divided these children into 2 groups according to the severity of CVA. 7 children were shown to have developmental delay in Group 1, while 14 children in Group 2, showing significant differences.

In group 1, only 1 child with risk factor of oligohydramnios has a neurodevelopmental delay in DDST. In Group 2, among the 14 children with developmental delay, the risk factors were found in 1 child with oligohydramnios, 2 children with breech, 2 children with twin, respectively. In other words, the results of this study showed that there is no definite relationship between 3 risk factors and neurodevelopmental delay. Although, these 3 risk factors are known to affect the occurrence of deformational plagiocephaly.^[3,5,6]^

As presumed by previous studies, brain growth in abnormally shaped skulls may have structural abnormalities manifested by significant developmental delays or deficits. Some cases were also found with cortical and subcortical abnormalities of synostotic plagiocephaly in neuroimaging studies.^[29,30]^ The findings of this study have showed that higher grade and severity of CVA in children with DP affect incidence of neurodevelopmental delay, which may support the previous studies. Although the exact underlying pathology and cause for our findings are not fully understood, 1 possible anatomical explanation is that the corpus callosum is positioned at a greater angle and shortened, and the height and height-width ratio of cerebellar vermis are found to be greater in children with DP,^[11]^ which implies that the higher the degree of head shape deformation, the higher the probability that developmental delay may occur. Therefore, it is possible to predict the occurrence of neurodevelopmental delay by measuring the scale of DP, CVA, and CVAI with simple method, which may provide an evidence for many pediatric rehabilitation physicians to consider further evaluation for children with DP to start rehabilitation therapy at an appropriate time.

This study has several limitations. First, because it is a retrospective study, there could have been limitations to collecting a comprehensive record of the patients. However, the total number of patients is 513, which is enough for statistical analysis. Second, we used only DDST to determine whether a child has a neurodevelopmental delay or not. Additional diagnostic tools for development such as BSID-III evaluation could have been applied to improve reliability. Finally, follow-up neurodevelopmental evaluations were not performed after the initial measurement.

## 5 Conclusions

In this study, 21 (4.09%) among 513 children with DP were found to have neurodevelopmental delay, which was affected by the severity of CVA and scale of DP.

It is found that the frequency of developmental delay was significantly increased in children with CVA more than 10 mm. Therefore, if possible, it could be suggested that reducing CVA to less than 10 mm with a conservative treatment or helmet therapy may help prevention of neurodevelopmental delay in children and doctors who take care of children with DP had better keep developmental delays in mild.

## Author contributions

**Conceptualization:** Dong Rak Kwon.

**Data curation:** Dong Han Kim, Dong Rak Kwon.

**Formal analysis:** Dong Han Kim, Dong Rak Kwon.

**Investigation:** Dong Han Kim, Dong Rak Kwon.

**Project administration:** Dong Han Kim, Dong Rak Kwon.

**Writing – original draft:** Dong Han Kim, Dong Rak Kwon.

**Writing – review & editing:** Dong Han Kim, Dong Rak Kwon.
